# “It Can’t Be Like Last Time” – Choices Made in Early Pregnancy by Women Who Have Previously Experienced a Traumatic Birth

**DOI:** 10.3389/fpsyg.2019.00056

**Published:** 2019-01-25

**Authors:** Mari Greenfield, Julie Jomeen, Lesley Glover

**Affiliations:** Faculty of Health Sciences, University of Hull, Hull, United Kingdom

**Keywords:** traumatic birth, birth trauma, choice, control, pregnancy, support, maternity

## Abstract

**Background:** A significant number of women experience childbirth as traumatic. These experiences are often characterized by a loss of control coupled with a perceived lack of support and inadequate communication with health care providers. Little is known about the choices women make in subsequent pregnancy(s) and birth(s), or why they make these choices. This study aimed to understand these choices and explore the reasons behind them.

**Methods:** A longitudinal grounded theory methods study involving nine women was conducted. Over half of the participants had a formal diagnosis of post-traumatic stress disorder (PTSD) and/or PND related to the previous birth. Interviews were carried out at three timepoints perinatally. These findings are from the first interviews at 12–20 weeks.

**Results:** From the first days of pregnancy, this cohort of women were focused on concerns that this birth would be a repeated traumatic experience. The women were deliberately searching out and analyzing information about their choices in this pregnancy and birth, and making plans which had two aims; firstly to avoid a repeat of their previous birth experience and secondly to avoid a loss of control to other people during the birth. The women considered a range of birth choices, from elective cesareans to freebirth. Some women felt well supported by those around them, including care providers, partners, friends, and family. Others did not feel supported and were anticipating conflict in trying to assert their birth choices. Many early relationships with healthcare professionals were characterized by fear and mistrust.

**Discussion:** If women who have previously experienced a traumatic birth become pregnant again, they have a strong desire to avoid a repeat experience and to feel in control of their birth choices. Access to robust information appears to help reduce uncertainty and arm women in their discussions with professionals. Similarly making plans and seeking to have them agreed with care providers at an early stage is used a way to reduce the risk of having a further traumatic experience. Implications for practice include supporting women in formulating and confirming pregnancy and birth plans at an early stage to reduce uncertainty and foster a sense of control.

## Introduction

The concept of choice in pregnancy is a difficult and contested idea. Women in the United Kingdom are all entitled to receive free perinatal care from community midwives from the point of conception until 2 weeks postnatally. Intrapartum care can be provided at home by midwives, or as a consultant-led service within a hospital setting. In most areas, women also have the choice of receiving midwifery-led care in a standalone or alongside midwifery led unit – however, these services depend on local availability. Local policies also dictate which women will be admitted to midwifery-led units in each area. It is unusual for most women to have met the midwives who care for her during birth.

Women can also choose to access private care perinatally. Private midwifery services are available through some hospitals and for-profit companies. Independent midwifery services are available from midwives who are self-employed. All private and independent midwives are trained in the same way as NHS midwives and are registered by and subject to the same practice standards as NHS midwives, however, they are not required to adhere to local NHS policies. Private midwives instead adhere to the policies of their employers, and independent midwives are required only to adhere to the standards set out by the Nursing and Midwifery Council (which all midwives are required to adhere to, whether working for the NHS, a private company, or as an independent midwife). Usually, if a private or independent midwife is hired, that midwife will deliver all perinatal care, including intrapartum care, to a woman. Occasionally private and independent midwives will work in a small team to deliver such care. Women using a private or independent midwife will pay directly for this service, and the charges for these services vary.

Some women also do not have midwives or obstetricians attend them during birth. This is a legal choice in the United Kingdom and is referred to as a freebirth. It is distinct from occasions when a woman gives birth without a professional present because the midwife was en route to a woman at home, or a woman was en route to the hospital when she gave birth.

Current UK policy advocates pregnant women being given choices, and being in control of decisions about their pregnancy and birth (NHS England, 2016). The benefits to women of having choices are well documented. These include maternal satisfaction (Mead, 2004) and personal control (Kightley, 2007). Conversely, a loss of autonomy in birth has been linked to lower self-worth, trust, self-esteem, and confidence (Edwards, 2004). Research, however, shows that this rhetoric does not always translate into women’s lived experiences (Jomeen, 2007), with the degree of real choice that women have often being limited to minor decisions (Edwards, 2004). Nevertheless, when women do make choices during pregnancy about their pregnancy or birth, the literature shows that they may be concerned with physical safety; factors, such as attitudes of family and friends, religious reasons, and confidence in the body’s ability to give birth also play a role in determining which choices women will make (Adam, 2016). The literature also shows that women often make decisions about the choice of birthplace before becoming pregnant, or during the first trimester (Murray-Davis et al., 2014).

Up to 30% of women in the United Kingdom experience childbirth as a traumatic event, with many consequently going on to experience some form of anxiety, depression, post-traumatic stress disorder (PTSD) following childbirth, or post-traumatic stress symptoms (PTSS) (Slade, 2006; Ayers, 2014). The literature suggests that the prevalence of postnatal PTSD in relation to childbirth is around 4%, but that over 18% of women in high-risk groups may develop PTSD postnatally (Yildiz et al., 2017). Cohorts who may be at higher risk of developing PTSD include women who have experienced a previous trauma in their lives, such as sexual assault or a previous traumatic birth, women who experience an unplanned operative birth with many interventions, women who receive poor care during birth, and women whose babies are born prematurely and/or require treatment in the neo-natal intensive care unit (Soet et al., 2003). Four trajectories for childbirth-related PTSD have been identified: resilience, (61.9%), recovery (18.5%), chronic-PTSD (13.7%), and delayed-PTSD (5.8%) (Dikmen-Yildiz et al., 2018). Dikmen-Yildiz et al. (2018) found that resilience was distinguished from other PTSD trajectories by less affective symptoms at 4–6 weeks postpartum. When affective symptoms at 4–6 weeks postpartum were removed from their model, less social support and higher fear of childbirth 4–6 weeks after birth predicted chronic and recovery trajectories. Poor satisfaction with health professionals is particularly associated with chronic-PTSD and delayed-PTSD and experience of further trauma and low levels of satisfaction with health professionals may compound these trajectories (Dikmen-Yildiz et al., 2018). If childbirth-related chronic-PTSD and delayed-PTSD is left untreated, it can continue to affect women for many years (Forssén, 2012). Consequences of traumatic birth include enduring mental health problems (Beck, 2004b; Forssén, 2012; Vignato et al., 2017), compromised maternal infant relationships (Nicholls and Ayers, 2007; Vignato et al., 2017) leading to adverse child cognitive development (Vignato et al., 2017), lower rates of breastfeeding (Vignato et al., 2017), poorer quality marital relationships (Ayers et al., 2006) and a negative impact on sex life (Vignato et al., 2017), concomitant depression in partners (Nicholls and Ayers, 2007), and can present a challenge to future reproductive decisions, with a higher proportion of women who have experienced a traumatic birth choosing not to become pregnant again (Fenech and Thomson, 2014).

It is already known that there are lower birth rates among those who have experienced a traumatic birth (Gottvall and Waldenström, 2002), and higher rates of elective cesarean section among those women who do have more children (Kottmel et al., 2012). Vignato et al. (2017) also found that experiencing PTSD from a previous birth may lead to women accessing less antenatal care in subsequent pregnancies. Little is known about other choices women make during pregnancy and birth, when they have previously experienced a traumatic birth, or what supports or hinders women in making these choices. The findings reported here form part of a longitudinal grounded theory study involving nine women who had previously experienced a traumatic birth. The study aimed to understand and explore the choices women made and the reasons behind them. Women were interviewed at three timepoints perinatally, in order to capture the different experiences of early pregnancy, approaching birth, and the immediate postnatal period. This paper presents the results from the first series of interviews (conducted between 12 and 20 weeks gestation); discusses the choices of the women in early pregnancy; and addresses the question “What choices do women make in the early antenatal period, when they have previously experienced a traumatic birth?”

The following definition of “traumatic birth” was used throughout the research:

“The emergence of a baby from its mother in a way that involves events or care which cause deep distress or psychological disturbance, which may or may not involve physical injury, but resulting in psychological distress of an enduring nature” (Greenfield et al., 2016).

## Materials and Methods

### Design

A constructivist grounded theory (Charmaz, 2014) approach was used, underpinned by feminist research principles (Stanley and Wise, 1993; Doucet and Mauthner, 2006). Data were generated from a series of three semi-structured interviews conducted during pregnancy and the early postnatal period. Theoretical models were developed at the conclusion of the three sets of interviews, but not from each set separately. Findings from this set of early antenatal interviews are therefore expressed as conceptual categories, and discussed as such rather than presenting a theoretical model as an outcome.

### Participants

Participants were recruited from April 2015 until September 2015 and all interviews took place between these dates. The study was advertised through a variety of online forums, including the Association for Improvements in Maternity Services, Doula UK, Association of Radical Midwives, local homebirth groups, Birth Choices groups, Independent Midwives UK, Mumsnet, Netmums, the Birth Trauma Association, International Cesarean Awareness Network, Birth Crisis, and Natural Mamas.

When women expressed interest in the research, they were sent an information sheet about the study. A follow up phone call was then arranged with the first author, at which point the information on the sheet was discussed. It was made clear to women that their information would remain confidential, and that they could withdraw from the study at any time, up until the analysis of the data was completed. If women wished to participate in the research, a consent sheet was completed, and signed by the woman. Consent was also revisited at the beginning of each interview. Women were recruited if they had previously experienced a birth which they described as a traumatic birth and which fitted the definition of a traumatic birth as given in the section “Introduction.”

In addition they had to have had a live baby from the traumatic birth; be between 12 and 20 weeks pregnant at the point they were admitted to the study; be over 18 years old, fluent in written and spoken English, and willing to participate. Women were excluded if their traumatic birth resulted in a stillbirth or neonatal death for two reasons. Firstly, the loss of the baby during a traumatic birth would mean that the woman was processing two major traumas, which were inextricably linked. It would therefore be impossible to ascertain how choices were influenced by the traumatic birth, as opposed to how choices were influenced by the death of the baby. Secondly, there was a high potential for revisiting these events in detail during the interviews to be re-traumatizing to interviewees. Women were also excluded if they planned to give birth outside the United Kingdom, due to the differences in maternity services internationally.

Sample size was determined by the concept of saturation (Charmaz, 2014). Interviews with participants were carried out immediately after recruitment. In total, 12 women were recruited to be interviewed; however, three withdrew before any interviews took place. The data set is therefore drawn from interviews with nine women, at which point no new data emerged, thus confirming saturation. As longitudinal grounded theory methods (GTMs) is a relatively rare methodology, the difficult issue of achieving saturation throughout the course of the research is not fully explored in the literature. In this research, the potential existed that saturation would be achieved at this timepoint, but then would not be achieved through interviews at timepoint 2 and 3. Details of the contingency plans that were made to accommodate this potential can be found in Greenfield (2018).

### Data Collection

To develop the semi-structured interview schedule, the research question was broken down into four areas. (What choices are women aware that they have? What choices are they making? What is their thinking about these choices? How do they feel about the choices they are making or have made?) Following a pilot interview, prompts were added to identify possible areas of choice including tests, scans, seeing professionals, diet, exercise, pregnancy groups, birth choices, and postnatal choices. The questions were designed not to be challenging, and not to demand justifications or explanations from the participant (Charmaz, 2014).

Data were collected through in-depth semi-structured interviews carried out by the first author, and undertaken via Skype. The decision to use Skype was made primarily to open up the study to women over a wide geographical area and facilitate recruitment. To allow the interviews to be participant led, an approximate idea of how long an interview might take was given but women were facilitated to lead on the length of time spent discussing each fundamental issue within the interview.

Despite birth histories not being specifically introduced as a topic by the first author, over the course of the interviews many women chose to disclose details of their previous birth(s), and how their traumatic experience continued to affect them during this pregnancy. Using Dikmen-Yildiz et al. (2018) categorization of trajectories, it is possible to hypothesize that the women who continued to experience significant symptoms during their current pregnancy were more likely to be displaying chronic-PTSD or delayed-PTSD trajectories, while the one woman who reported no symptoms during her current pregnancy may have followed a resilience or recovery trajectory. As not all the women included in the study had a formal diagnosis in relation to their traumatic birth, caution should however be exercised in assuming this hypothesis to be accurate.

### Data Analysis

Analysis began as soon as the first interview was completed, and continued alongside data collection throughout the research. The process described by Charmaz (2014, 2006) was followed. All interviews were conducted and transcribed by the first author. Transcripts were then anonymized, with identifying names and locations being removed. All women were offered the opportunity to choose a pseudonym, and the first author chose a pseudonym for those participants who did not want to choose one themselves. Each transcript was re-read several times, and then coded line by line. Coding enabled analytic categories to emerge, previous ideas to be refined and/or discarded. These developments were noted in memos, attached to the electronic documents as comments. Further theoretical ideas which emerged during this process were noted in a reflexive diary, for later consideration.

Simultaneous data collection and analysis allowed the validity of the categories to be checked through discussion with participants, providing an initial quality check for the research. This process also allowed the refinement of emerging connections between categories. A matrix was constructed, which combined quality checks proposed by Charmaz (2014); Stanley and Wise (1993), and Kelly et al. (1994). The matrix comprised of the following criteria: credibility, originality, resonance, usefulness, and positive impact on women’s lives. The research was evaluated by comparison with the matrix, to ensure its quality.

### Reflexivity

The first author drew on a large body of both GTM research, and feminist literature to develop an approach to reflexivity for this study. In this research, reflexivity had a number of complementary purposes – introspection, intersubjective reflection, and social critique. Finlay’s (2002) categorization of approaches taken to reflexivity was utilized to produce a map of how these elements fitted together to form the reflexive approach of the research (further details of which are given in Greenfield, 2018).

A central part of the reflexive approach adopted in this research is acknowledging the position of the researcher. This statement outlines the first author’s personal experience, as relevant to the research:

The first author is a woman, and also a mother. She has experienced pregnancy, labour and birth within the UK, and through this has had a range of personal relationships with HCPs. The first author also works as a doula, a breastfeeding counsellor, and an Infant Massage instructor. In approaching this research, the first author therefore brings her own personal, professional and social positions to the research. This may have influenced her interest in women’s experiences of birth, and therefore the formation of the research question.

Birks and Mills (2011) series of consciousness-raising questions were adapted to incorporate these three elements of reflexivity. The adapted questions were asked on a regular basis to aid thinking about power differentials that might exist in the research relationship and to ensure a conscious, ongoing commitment to participant-driven research. Discussions with the co-authors also aided reflexivity.

## Results

### Demographic Data

**FIGURE 1:**
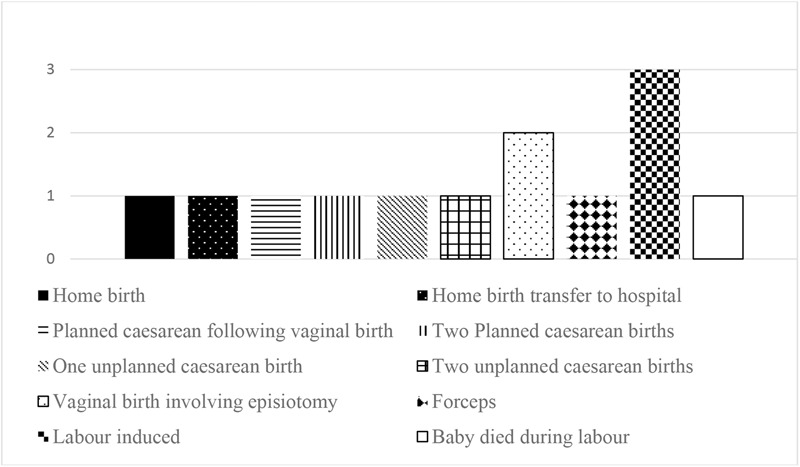
Previous birth experiences.

All the women recruited to the study were White British, and all described their sexual orientation as heterosexual. The women’s ages ranged from 24 to 43 years old at the time of recruitment. None of the women described themselves as having a disability. Five of the women had a formal physical or psychological diagnosis in relation to their traumatic birth. The women had varied fertility and birth histories, ranging from one previous child to three. Women’s previous birth experiences are shown in Figure 1 (total is greater than number of women due to multiple births).

The number of previous pregnancies (min = 1, max = 4, average = 2) showed that some of the women had experienced multiple losses before this pregnancy. For most of the women, the traumatic birth had been experienced with their current partner, but for two of the women the current pregnancy was the first child they had with this partner. The gestational age at point of initial interview ranged from 12 to 20 weeks (average = 17.2 weeks).

The women were not asked for specific details of the previous traumatic birth(s). This was in part an ethical decision made to avoid retraumatizing women during pregnancy, as some literature suggests that reliving previous trauma through psychological debriefing can lead to increased PTSD under some circumstances (Rose et al., 2002). Trauma is also a subjective experience, and therefore relying solely on women’s accounts of why they experienced their births as traumatic would not produce robust data within the confines of this study.

The exception to this relates to the participant whose baby died during labor. Women were excluded from the study if their traumatic birth had involved a stillbirth, intrapartum, or early neonatal death. After consideration, this participant was admitted to the study as that birth, while having a very sad outcome, was not her traumatic birth. In this case, her birth history was discussed during the initial recruitment interview, in order to establish whether she could be accepted into the study. As the pregnancies that would be discussed in detail during the interview were likely to be the current pregnancy and the pregnancy that resulted in the traumatic birth (which was not the birth where her baby died), the risk of re-traumatizing her through prolonged discussion of the death of her baby was felt by the authors to be reduced. Further details of each woman’s previous history are given in Greenfield (2018).

### Conceptual Categories

The conceptual categories emerging from these interviews in the early antenatal period centered around the ways women were dealing with their previous traumatic birth, and their efforts to ensure that this pregnancy and birth were different to the previous one. The conceptual categories are shown in Table 1.

**Table 1:** Categories from early antenatal interviews.

Categories
Feelings about being pregnant and “the bump”
Gathering and analyzing information
Making plans for this time
Choice and control
Support
Postnatal decisions

From the conceptual categories, several inter-related categories emerged as dominating women’s thoughts and experiences in the early antenatal period, and are examined below.

#### Feelings About Being Pregnant

The women interviewed experienced a range of mixed emotions about their pregnancies in general, but every woman expressed anxious feelings – “*I’m … quite anxious … quite worried*” (Lea). These feelings ranged from being “*apprehensive but, but you know excited*” (Victoria) to being “*very very nervous*” (Alice). Specifically, this group of women had very negative emotions when thinking about “*the delivery aspect*” (Taylor), if they could indeed think about these issues at all.

“*I still can’t really think about … erm, birth side of things yet*” (Lea).

Not only was birth the sole focus of real concern, the women interviewed shared one very specific worry about the birth, which quickly became the main feature of the early pregnancy interviews:

“*my fears are that … the, my labour is going to end up being, how it, how it was last time*” (Taylor).

For pregnant women who have previously experienced a traumatic birth, thinking about the impending birth in an early stage of pregnancy appears to provoke a great deal of anxiety and worry.

#### Gathering and Analyzing Information

In response to this overriding concern about the forthcoming birth, every woman interviewed talked about information she had gathered about pregnancy and/or birth choices. Even at this early stage of pregnancy, women had deliberately gathered a huge amount of information from diverse sources. The majority of this information related to birth choices, rather than to the pregnancy.

Most women were offered medical opinions and information by “*my midwife*” (Lea), including both “*independent midwives*” (Quinn) and NHS midwives, “*the head of midwives*” (Alice), “*GPs*” (Quinn), “*the [NHS] consultant*” (Alice), and “*private consultants*” (Rachel). Where women had sought this information out, and health care staff had made the time to talk to them, women were very appreciative – “*It was very reassuring to me*” (Quinn). Another important source of medical information was the meetings some women had following their traumatic births, either with the consultant to discuss follow up care, or through a Birth Afterthoughts or “*Birth Reflections*” (Taylor) service “*the reflections appointment was … erm, amazing, informative. I found things out, about my first um, labour and, birth, that I didn’t have … a clue about.*” (Taylor).

Women were sometimes aware of not having been given all the information, or the correct information, or of having information presented to them in a less than optimal way in their previous birth by medical professionals. In one extreme case, a woman’s first baby had sadly died during labor. She felt that she had been misled by the healthcare professionals around her during this birth. At the time,

“*the reports that the Trust wrote about … declining of … continuous monitoring [they said] that caused his death … And it wasn’t until [first baby]’s post-mortem came back at 16 weeks postpartum, that … … said categorically that … even if he’d have been born, by caesarean, while I was in labour, he wouldn’t have lived because, his brain was so hypoxic*” (Luna).

This experience led her to treat any information given by medical professionals with a degree of mistrust throughout her subsequent pregnancy and the (traumatic) birth of her daughter, which had continued into the current pregnancy – “*they just lie*” (Luna).

Additionally, some women were also given information they believed to be incorrect during this pregnancy

“*the midwife … she was like, so, if your scar does rupture, which I said, yeah but that, y’know, that’s a less than 1% chance, she was like, yes it will be catastrophic and you will both die, you won’t make the ambulance, and I was like, not entirely sure that’s entirely accurate ...*” (Becca).

These experiences of being given incorrect, partial, or incomplete information in previous and/or current pregnancies led to some skepticism about medical information and opinions given this time, with women looking to gather information from other diverse non-medical sources.

A number of the women interviewed talked about using written materials to gather information about their pregnancies and birth choices. One interviewee’s main source of information was “medical research literature” (Rachel). Other women preferred books as a source of information and read a huge variety of them. For most of the women who used written materials as an information source, this information was supplementary to something else.

Several women recounted gathering information by watching TV programs which show different types of birth experience, but talked about selecting programs “*through careful consideration*” (Luna), and avoiding others

“*I don’t watch … One Born Every Minute, cos as far as I’m concerned it’s like a horror film*” (Luna).

Becca described how she spent time “*shouting at One Born Every Minute*” (a television program showing births in hospitals), and described this as part of the way she educated her partner about her views and experiences of birth.

Other women chose to physically attend groups and as a way of obtaining information found them “*helpful*” (Victoria). Some women were not able to attend groups due to other demands of life

“*I’ve got four kids under 6 … I have to schedule in time to brush my teeth, y’know*” (Becca)

while others had a general disinclination toward groups. Some women chose deliberately not to attend groups, feeling strongly that they were not a source of information that they wished to access. Luna described avoiding groups because “*outside influence really affects … my balance*,” something which she also said about an offered and declined meeting with a consultant. For Luna, being able to select the type of information she exposed herself to was critical. Making these choices involved a pre-judgment about what information would be likely to come from a specific source, and a choice then to receive information from that source or not. This strategy was used to some degree by several women.

Although women were using technology and the media to gather information, they were not relying on it as a source of completely factual information:

“*of course that’s just what the Internet says, just opinion not fact*” (Luna).

Instead, during the interviews the women displayed an understanding that some accounts were biased, and had a healthy skepticism about their wider application, or relevance to the experience of the participant;

“*I’m not really too keen on reading much online because I think … everybody has such a different story don’t they. You can’t take from other people’s stories. Or the information changes*” (Alice).

Rather, women appeared to be gathering a large amount of information, and then sifting through it to see what was applicable to them. What women said they were looking for was somewhere to go for answers tailored to their individual circumstances

“*I felt I had questions, more relevant to me*” (Victoria).

One consequence of this view of non-medical sources of information as being potentially biased, and generalist information rather than tailored individually, was that women then sometimes extended this view to information given by medical professionals too

“ *[the midwife said] they [the Obstetric team] assume you should go and give birth in that hospital, because you’ve seen the consultant there. Which was… I’m thinking well that’s not true either because, I, I, I’m entitled to get a [second] medical opinion and could go elsewhere anyway*” (Taylor, 1).

Women did not unquestioningly accept that a medical opinion was correct. This seemed to emerge partly from the experience of sifting and analyzing other sources of information.

#### Making Plans for This Time

**FIGURE 2:**
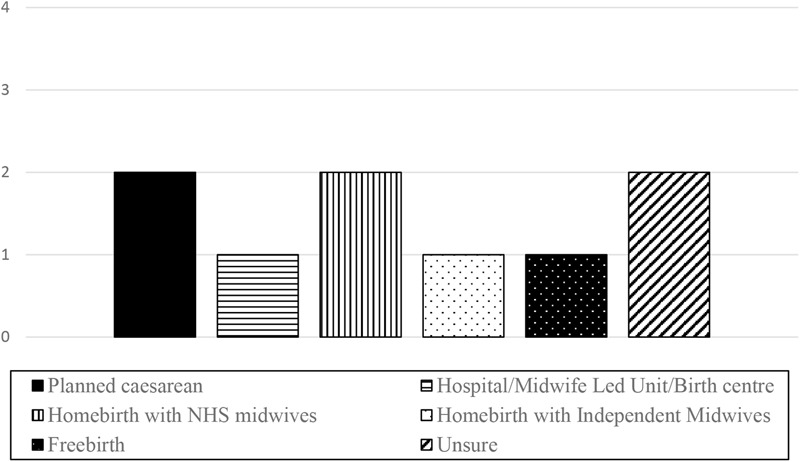
Birth plans at 12–20 weeks of pregnancy.

Even at this early stage in pregnancy, women were concerned with making plans for the forthcoming birth. The information they had gathered directly informed their plans for this birth. Figure 2 shows how women planned to give birth at the time of the interviews.

##### The act of planning

For some, the birth planning had started before they conceived

“*I did also see a GP a few months before I started trying to conceive … then I said to her oh well I’d like a home birth*” (Quinn).

The planning for birth continued during early pregnancy. For some women, the choices they made about tests during early pregnancy were very connected to their later plans, especially if they felt that choices they made could impact their choices about birth. This affected both whether women accepted or refused various services and tests that were offered:

“*I … have agreed to erm thyroid testing every 10 weeks … That’s not something that will exclude me from a home birth, that will not exclude me from, you know, any of the things I want to do*” (Luna).

Some of the women had distinct preferences at this stage about how they would like to give birth

“*Electing for a caesarean is a very conscious choice, it’s not really a choice when the choice is between having elective caesarean or risking permanent incontinence*” (Rachel).

But for most, there were elements of uncertainty

“*At birth. I’m, I’m UNsure. I don’t know [whether she would like a homebirth with NHS midwives, independent midwives, or a freebirth]*” (Luna, her emphasis).

For two of the women, not planning was important. They both expressed strongly that, although they had some preferences in how they birthed, they were refusing to plan. At the same time, both were making decisions about care. For them, actively choosing not to plan was important. Although their birth plans were very different, what they had in common with each other was that both had experienced two traumatic births previously. When asked about why not having definite plans was important, one answered

“*So I’m not setting myself up to fail later on*” (Becca).

##### Content of plans

The women interviewed were planning a wide range of different ways to give birth, including freebirth, homebirth with independent midwives, homebirth with NHS midwives, hospital births, medically indicated cesarean sections, and non-medically indicated cesarean sections. No two plans were alike in terms of choice of birth. But what their plans did have in common at this early stage of pregnancy was a list of things that the women absolutely did not want.

What was viewed as unwanted was different for every woman. For some, a specific way of birthing was strongly not wanted. For Luna, a cesarean

“*would never be, that would never be my choice.* Never” (Luna),

while for Rachel “a vaginal birth” for this baby was definitely not something she wanted. For others, specific interventions were not wanted. Taylor was extremely clear that

“*absolutely desperately, I do not want pethidine*” (Taylor),

while for Victoria, “avoid[ing] an episiotomy” (Victoria) was the main focus of her plans this time, and for Luna

“*there are so many options that I will take over an induction again … for this* baby” (Luna).

For some, it was about who was there at the birth – “*midwives attending*” (Halle), or what specific people did during the birth – “*no-one else is doing it [caesarean] this time*” (Alice).

These extremely strong wishes to avoid certain things at all costs frequently came directly from the experiences women had during their traumatic birth(s). Ensuring that this birth was “*not like last time*” (Alice) was the driving force behind the plans that the women were making. It drove how women searched for and analyzed information about their previous births, and about their current choices. By planning to refuse specific unwanted things, women sought to avoid a repeat of their previous experience

“*and I just don’t want to get in that state again … So I just think I need that control over the situation*” (Lea).

Many of the women acknowledged that planning for birth was difficult, because there are many unknowns

“*you have to go with how it, goes along*” (Alice).

This negative planning (planning not to have certain births, interventions, and so forth) was a way women could regain control over their birth experience

“*I need to be in control next time, because they took all the control away from me and left me very vulnerable and I need to have that control this time and my wishes respected in order for it to be a good experience. So that’s why there’s gonna be quite rigid birth plans*” (Quinn).

#### Choice and Control

Most women talked about the choices they were making. They saw exercising their choices as the way to achieve the goal of having a birth that was different from last time

“*… the first time round I’d have done what I was told. I’d have just done what I was told and the impact of that is I’m saying do you know what, no. This, everything is my choice. It’s my choice. It’s my baby my body my birth. I don’t want to do that, that’s not going to happen this time.*” (Taylor).

Women were very deliberately making plans which they felt protected them best against any loss of choice or control they had experienced in their traumatic birth. This was especially noticeable when they perceived an individual or individuals (rather than the naturally occurring events of birth) had removed that control from them:

“*I’d like more, control … I put a lot of trust in the team [last time] … and a few of them let me down … making some distance a bit, this time*” (Victoria).

Losing choice or control due to natural events outside of anyone’s power was upsetting to the women, but did not result in the same level of detailed planning as the previous removal of choice or control by another person did.

For many of the women, exercising their right to choose was not described by them as a simple matter of making a decision and communicating this. Even at this early stage of pregnancy, many women were anticipating having to argue or even fight for their right to make the choices they wished to make. At this stage in pregnancy, few of the women were expecting that their right to make choices would be supported.

#### Support

Women’s desire for support for their choices for this pregnancy was extremely high. It was mentioned multiple times in every interview, without exception. Often, discussions about what support a woman wanted this time stemmed from, or led to, comments about the support or lack of support she had received during her traumatic birth. Women wanted different support from different people involved in their lives.

##### Support from midwives

At this early point in pregnancy, all the women were being offered care by midwives. For most of the women, midwifery care at this stage was provided by NHS Community Midwives, for a small number, the care was provided by independent midwives. Yet in these first interviews, midwives seemed strikingly absent in what the women talked about.

In some cases, this absence was due to what women wanted from their community midwives. Some women who wanted consultant-led care described midwives as the gatekeepers to that care, particularly if the woman wanted a non-medically indicated planned cesarean birth. The support women wanted in this situation was a referral, and not a lot else. Once a midwife had done what the woman wanted in terms of referring her, there was a perception that the midwives had no further role. In the pilot interview, the participant, who was having an elective cesarean birth said:

“*[I] say to my colleagues, ‘Just going to see the chocolate teapots’ when I’m off to the midwives*” (pilot interview).

No other data from the pilot interview was included in the analysis, but this one statement was expressed so strongly that it was included as it indicated support for this finding. Although this was the most negative way a woman wanting a non-medically indicated cesarean birth described her midwives, it was a sentiment which was shared by other women;

“*I think I’ve almost like bypassed the midwife*” (Taylor).

For these women, getting the all-important referral was very much on their minds at this stage of pregnancy, as it was the first step in their journey toward securing the birth they wanted. Some of these women also had concerns at this early stage of pregnancy that they might be “pushed” by midwives to a less medical choice, for example, not having an elective cesarean section, or using a birth center instead of hospital labor ward.

“*I’ve been offered an appointment [with the community midwives at a VBAC clinic to discuss birth choices] … I will be going even knowing that I won’t be a candidate for it, a vaginal birth. … because it’s useful for me to erm, document it somewhere the reasons for the choice that isn’t a choice.*” (Rachel).

Conversely, women who didn’t want consultant-led care described feeling that they had to be firm with the midwives to avoid a referral. They expressed concerns about midwives “pushing” them down a more medical route – for example into a hospital birth rather than a homebirth, or into an attended birth rather than a freebirth:

“*they’ll try anything they can to sway me the other way … kind of, more medicalised birth I guess.*” (Becca).

Other women felt pushed by midwives into tests they didn’t want, the results of which might lead to the women being further pressured into birth situations they didn’t want “*Erm … the nurse in the clinic, kind of did a bit of a sneaky thing what, which was, we would like to do, erm, er, an HbA1c. And I know you’re not keen, but, can we do it anyway? And I sort of went, ^∗^sigh^∗^, you know what? I just want to go home, just take my damn blood and do it. And of course, it all came back fine, thank god. But, I did feel pushed into that*” (Luna).

The removal of choice that happened in this instance, and the lack of support shown, had further reaching consequences than just whether that test was carried out or not. Luna described how this incident contributed to her deteriorating “trust” (Luna) with not just this midwife, but “the lot of them” (Luna).

For women choosing either a birth with less monitoring or interventions than might be recommended by local policy, or wishing to choose an elective cesarean, the fear of being encouraged or forced down a path they did not want to take led to a sense of conflict with the midwife or midwifery team, and also affected women’s emotional state profoundly:

“*that anxiety became bad after I spoke to … she kept talking about a hospital transfer and things like that and it just freaked me out and that really sort of I cried for about three days after that phone call.*” (Quinn).

This sense of conflict may not have been based solely on the actions of a midwife involved in this pregnancy. Rather, it seemed to come from previous negative experiences, which left women feeling vulnerable in subsequent pregnancies. This is not to imply that the fear felt by women was unreal, or baseless – rather it was a fear based on their personal experience of a previous midwife, midwifery team, or doctor. This fear led women to approach antenatal appointments as though each might be a battle to assert their rights, preparing for each appointment carefully, marshaling the information they had collected as a pre-emptive defense. The combination of fear and vulnerability also led to some women actively avoiding or keeping midwives at a distance, relegating midwifery care to a peripheral role:

“*I feel a little bit more jaded and a bit wiser about things, and I, as I say I, I’d, I’m happier keeping my distance.*” (Victoria).

On the other hand, where a relationship with a midwife was good, this was highly valued by women. At this early stage of pregnancy, being listened to was valued highly, alongside the midwife respecting not the choices a woman was making *per se*, but the woman’s right to be the one who made the choices

“*Very supportive community midwife.*” (Luna).

Equally valued was having a midwife who a woman felt really understood her experience of trauma

“*my community midwife is absolutely lovely … Um, and she had a lot of difficulty bonding with her son. Cos she had a difficult birth … And she had postnatal depression. So she understands from a midwife point of view but also from a, you know baby point of view … It’s really nice to have someone who gets it.*” (Lea).

One of the women involved in the research received all her care from independent midwives at the point of the first interview. For this woman, the relationship with the midwives was crucial:

“*it made me think you know what, these women know what they’re doing I can trust … and that sort of helped me feel like I could do it again*.” (Quinn).

##### Support from partners

At the time of the first interview, all the women involved in the research were in a heterosexual romantic relationship with the father of the baby they were currently pregnant with. All the women desired the support of their partner for their birth choices. Some felt they had it

“*and he’s, um, supportive to the point where it can be annoying.*” (Becca).

While other women felt less supported in their choices at this stage of pregnancy;

“*my husband with the best will in the world erm … wants to support me and I erm, and I, I am sure he, he does try, but … he’s just got a different angle*” (Victoria).

Some of the women had worked hard to ensure their partner understood what they needed from them. This was particularly the case for the two women who were pregnant to partners they had not had a previous biological child with. In these cases, the women described how their view of pregnancy and birth were different to their partner’s views

“*he’s still in that mindframe that if a professional said that sort of thing, they’re right, they know best, they’re the experts*” (Taylor).

“*he owes his son’s life to doctors, whereas …in my head, they’re kind of interfering with what has caused the problems that I had …our experiences of childbirth are very different, um, and I was really scared that the choices that I make in this pregnancy would scare him.*” (Becca).

These two women worked hard to educate their partners about their previous experiences, because it was important to them that their partners understood their traumatic experiences. Interestingly, the two women were hoping to have very different births at this stage of the research (elective cesarean birth and homebirth), but the journey they describe going through with their partners was very similar.

## Discussion

If women who have previously experienced a traumatic birth become pregnant again, they may be dealing with complex feelings about their previous birth experience, have a strong desire to both avoid a repeat experience and to feel in control of their birth choices. It is usual for pregnant women to experience mixed feelings, including anxiety, during pregnancy (Cantwell and Cox, 2003). However, the intense focus on the birth in early pregnancy separates these women’s experiences from the experiences of other women in early pregnancy, who often do not “dwell on labor” until later in their pregnancies (Jomeen, 2010, 147). In this study, the women’s overriding focus from very early pregnancy was on preventing this birth from being “*like last time*” (Alice), and to do this they felt they needed to be in control of the choices surrounding their birth. The loss of control they experienced in their traumatic birth(s) had damaged their trust in those supporting them, hence all subsequent behaviors and navigations were designed to ensure control in this pregnancy. This finding is consistent with a large body of existing literature, which identifies a loss of control as a predictor of traumatic birth (Gatrell, 2005; Kitzinger, 2006; Slade, 2006; Jomeen and Martin, 2008; McKenzie-McHarg et al., 2015; Hollander et al., 2017). This paper will therefore discuss some of the key ways in which women sought control, and then examine the practice implications for care providers working with this cohort.

### Information Provision

All pregnant women need access to good quality information about pregnancy and birth, available in a variety of formats (McCants and Greiner, 2016). Pregnant women who have previously experienced a traumatic birth are no different, but they may need more specialist information to assist their understanding of their previous experience and may use the information in different ways (Beck and Watson, 2010). Intense information gathering by this cohort has been noted by Thomson and Downe (2010) and by Beck and Watson (2010). Thomson and Downe (2010, 105) identified several purposes to this activity; both “resolving the past” traumatic birth, and preparing for the uncertainty of the forthcoming birth. Beck and Watson (2010, 245) observed that information gathering and birth planning were part of women’s “strategizing” to remain in control of this birth, in order to avoid a repeat traumatic experience. In this study, it was notable that women also used information to check the accuracy of information given by health care professionals, to inform their planning, and to arm themselves in their attempts to negotiate care. The links between trust and information seeking are clear – unlike other populations, who tend overall to view care providers as a source of accurate, trustworthy information (Fainzang, 2015), this cohort of women felt the need to be independently informed due to their mistrust of care providers, and to validate care provider advice.

### Finding Care Providers Who Can Be Trusted

Most of the women in this study had entered their previous birth assuming that trusting care providers was an appropriate behavior. The traumatic birth had then served as what Holmes (1981) describes as a “strain-test” for the trust invested in the relationship. In strain-test situations, one individual is highly outcome dependent on another person, but the actions that would promote the individual’s own interests differ from those that would benefit the other. In the case of traumatic birth, the birth itself had provided the strain-test, but it was the actions, inactions, or words of the care providers during that crisis that had resulted in the women experiencing a loss of trust. Trust had been lost not only in the specific individuals caring for them, but the women had concluded that trusting either care providers as an entire group, or specific sub-groups such as midwives or obstetricians, was unsafe.

For the women in this study, the only way to feel they had regained that safety was to remain firmly in control of their birth, and they employed a number of strategies to achieve this. The result of this situation is that the women entered their current pregnancy mistrusting the care providers who were offering care during the pregnancy. The expectation of unsupportive care and embattlement had diverse consequences, from women seeking to avoid antenatal appointments, to women feeling unsupported by care providers, and distressed by their exchanges with them. This finding is consistent with the existing literature; women who have previously experienced a traumatic birth may experience a lack of trust in those around them during a subsequent pregnancy (Beck, 2004a). The literature also shows that feeling “connected” to and having trust in the professionals caring for them is an important determinant of a subsequent birth being a positive experience (Thomson and Downe, 2010).

A pre-requisite to the rebuilding of trust involved finding a care provider who would acknowledge the previous traumatic experience at an early stage in the current pregnancy. For women who have had a previous traumatic birth, rebuilding trust with care providers can only begin with an understanding that trust was previously given and was betrayed, or even abused in order to coerce women (Gould, 2004).

### Birth Information Review Meetings

Pregnant women who have had a traumatic birth may need to gather information about their previous birth, before being able to consider information about their current pregnancy. This does not fit the current NHS model of antenatal care, which focuses to a large degree on the current pregnancy (NHS England, 2016). Some women involved in this research had the opportunity to gather the information they needed about their previous birth(s) through a meeting with a senior midwife, often referred to as a Birth Reflections or Birth Afterthoughts meeting. These meetings are generally offered as a standalone appointment, usually held somewhere other than the hospital in which the birth occurred, in which the medical notes and the woman’s own account of her experience are discussed. These meetings have been found to be of value to all women postnatally, when conducted in the immediate postnatal period, as a way of validating their experiences (Baxter, 2017). Baxter also found that “women with a high Impact of Events Score (IES) are more likely to want to talk following their birth experience,” which supports the view that such meetings are even more valuable for women who have experienced a traumatic birth (Baxter, 2017, 11). In this study, women were accessing these meetings at a different timepoint to the women in Baxter’s study, as they were pregnant again, but they showed a similar need to talk about their previous birth experience. Those who attended such meetings generally found them useful, as some of their questions about what had happened, and what could be done differently in this birth, were answered. However, in some cases for the women in this study, these meetings served to increase their lack of trust in care providers. In addition to these meetings, women often continued to look for further information, and then had limited access to care providers to discuss what they had found, which raises questions about the purpose of these clinics and whether this is an ideal fit for this particular group of women.

Debriefing for women who have experienced a traumatic birth is controversial, because of the links to PTSD. It is widely acknowledged that some women develop PTSD following childbirth (James, 2015; McKenzie-McHarg et al., 2015), and also that it is underdiagnosed (O’Donovan et al., 2010; Yildiz et al., 2017). The result is that women presenting for a debriefing following a traumatic birth may or may not also have PTSD, and evidence for psychological debriefing following other types of traumatic events shows it can lead to increased PTSD under some circumstances (Rose et al., 2002). UK guidelines for PTSD treatment therefore explicitly state that debriefing should not be used (NICE, 2005). Whether this should apply to the midwife-led debriefing detailed above is difficult to determine, partly because of huge variability in what midwife “debriefing” comprises (Steele and Beadle, 2003; Thomson and Garrett, 2018). Recent work by Sheen and Slade unpicks some of these issues, arguing a case for targeted debriefings, and specifying what these should involve. Their work is however primarily concerned with whether debriefing “reduce[s] symptoms of PTS or depression,” and/or whether it is “efficacious … for women who experienced a traumatic birth” (Sheen and Slade, 2015). For the women in this study, engaging in a birth reflections meeting was not primarily intended to result in psychological or emotional improvement, but was used by the women as a tool to help them prepare for their forthcoming birth. An evaluation of one “listening clinic,” specifically established to “discuss preferences for birth, to debrief about a previous birth, or both” found that the service was valued by women, and worked well as a complementary service to standard midwifery care (Stalberg, 2017). The findings from this research support the findings from Stalberg’s research that this service is different to debriefing, and is of real value, especially for women who have experienced a traumatic birth. As no evaluation of birth reflections meetings as a generic tool for supporting a subsequent pregnancy have been conducted, it is difficult to state whether this is a generally effective tool. Further research to establish the utility of such interventions would be advantageous.

### Practice Implications

The midwife caring for a woman who has experienced a traumatic birth and has lost trust in midwives faces a difficult job. Without a trusting relationship between woman and midwife, the midwife’s ability to provide a caring role is limited (Gould, 2004). Hunter (2006) describes the “additional emotional work” midwives have to engage in when interactions with the women they are caring for are “difficult.” She goes on to define the consequences for midwives of dealing with these “emotionally difficult situations,” which included midwives feeling “overwhelmed and out of my depth,” and notes that this situation also has an effect on mothers, as some “midwives manage their emotions by self-protective strategies, such as professional detachment, distancing and task orientation … [which] inevitably affects the quality of care that women receive, and may explain the uncaring attitudes” (Hunter, 2006, p. 319).

For a trusting relationship to be established, the midwife needs to understand and acknowledge the previous traumatic experience, alongside any breach of trust that previously occurred. The midwife must give this acknowledgment of the past, and any abuse of trust this entailed while at the same time, attempt to build trust with a woman who may present as highly suspicious. Only once these two undertakings are achieved can the midwife begin to effectively offer care. Subsequently, the midwife must continuously engage in strategies which enable the woman to feel in control, if the relationship is to continue as a positive and trusting one.

If the relationship is then to develop, all care providers involved in a woman’s care must then employ strategies that promote her sense of control. In the current models of care employed within the NHS, it is likely that women will meet multiple midwives throughout the perinatal period, and potentially other care providers, such as consultants, as well. Risks therefore exist that the trust-promoting acknowledgment of the traumatic birth provided by one midwife may be undermined by an insensitive interaction with another care provider.

Similarly, all those who are involved in the care of a woman who has previously experienced a traumatic birth may need to employ strategies that promote the woman’s sense of control, as an experience of disempowerment by one care provider may have concomitant impact on the woman’s trust in others.

Continuity of carer has been found in other literature to promote to a trusting relationship between a woman and those providing maternity care to her (Thomson and Downe, 2010), and the early findings from this study appear to confirm that this is advantageous. A woman who has previously experienced a traumatic birth who does not receive continuity of carer may experience further damage to her ability to form a trusting relationship with her care providers. This poses a challenge to the current UK maternity context, where midwifery is not organized so that continuity of carer is a common experience for most women.

### Limitations of the Study

Although the study met its aims, some limitations have been identified. Firstly, as with much qualitative research, the overall number of participants was small. The effect of the sample size is that the findings may not be able to be generalized to the entire population of pregnant women who have previously experienced a traumatic birth. Additionally, while this research was not intended to provide a representative sample, it should be noted that the participants were all white, all identified as heterosexual, and all were living with a partner at the time of recruitment. Targetted recruitment to include a more diverse sample was carried out, and the research was advertised on LGBT doula forums, and within the “mothers of color” pages within Mumsnet – however no participants at the right stage of pregnancy came forward from within these groups. This means that the experiences of all women are not captured in this research. It may be that women belonging to other ethnic groups, lesbian or bisexual women, and single mothers may have a different experience of pregnancy subsequent to a traumatic birth.

The research question was formulated to examine the choices that women were making. In the development of the interview schedule, it was acknowledged that women can only make choices they are aware of being available to them. In the interviews themselves, it was quickly apparent that not all the participants were aware of all the potential choices that were available. Women’s ability to make choices can, however, be limited by factors other than knowledge. This arose as an issue during the interviews, in that Rachel wished to have her cesarean section carried out by a private obstetrician, but was unable to access this due to a loss of private medical insurance through her partner’s employment, and Luna wished to employ an independent midwife, but did not have the financial means, independent of her partner, to do so.

## Conclusion

When women who have previously experienced a traumatic birth become pregnant again, they are likely to focus on ensuring that this birth is different to their previous experience. Correctly identifying and supporting these women in the early antenatal period is a necessary preventative measure to decrease the likelihood of a further traumatic experience. Effective healthcare can only be provided to this cohort of women following the development of a trusting relationship between the woman and the care provider. Continuity of carer may be advantageous to allow such a relationship to develop. The women’s focus on ensuring that their birth experience is different may lead to a need to ensure they feel in sole control of decisions, and they may employ specific strategies to do this, through their birth plan. Conversely, if women have experienced multiple previous traumatic births, they may be very resistant to formulating written plans, as a self-protective mechanism, while still retaining the desire for control of those choices. In either case, sensitive and responsive individualized care by care providers is likely to support women.

When a woman who has previously experienced a traumatic birth becomes pregnant again, her overriding desire is that the birth will not “*be like last time*” (Alice). This research shows the importance of early antenatal care, and the strategies that can be employed by care providers to help women achieve a different birth experience.

## Ethics Statement

This study was carried out in accordance with the recommendations of the ethical guidance of the University of Hull Ethics committee. The protocol was approved by the University of Hull, Faculty of Health and Social Care Ethics committee. All subjects gave written informed consent in accordance with the Declaration of Helsinki.

## Author Contributions

MG identified the research topic. All authors contributed to the development of the research design, manuscript conception, drafting and development. MG collected all data and undertook the analysis and interpretation under the supervision of JJ and LG. MG, JJ, and LG contributed jointly the article.

## Conflict of Interest Statement

The authors declare that the research was conducted in the absence of any commercial or financial relationships that could be construed as a potential conflict of interest.
